# Investigating the Specific Effects of an Online Mindfulness-Based Self-Help Intervention on Stress and Underlying Mechanisms

**DOI:** 10.1007/s12671-017-0867-y

**Published:** 2017-12-18

**Authors:** Jenny Gu, Kate Cavanagh, Clara Strauss

**Affiliations:** 1 School of Psychology, University of Sussex, Falmer, East Sussex, BN1 9QH UK; 2 Sussex Partnership NHS Foundation Trust, Hove, BN3 7HZ UK

**Keywords:** Mindfulness, Self-compassion, Stress, Mechanisms, Self-help, Online, RCT

## Abstract

Previous research examining the effects of mindfulness-based interventions (MBIs) and their mechanisms of change has been hampered by failure to control for non-specific factors, such as social support and interaction with group members, facilitator contact and expectation of benefit, meaning that it remained possible that benefits of MBIs could have been attributable, perhaps entirely, to non-specific elements. This experimental study examined the effects of a 2-week online mindfulness-based self-help (MBSH) intervention compared to a well-matched classical music control condition and a waitlist control condition on perceived stress. This study also tested mindfulness, self-compassion and worry as mechanisms of the effects of MBSH versus both control conditions on stress. University students and staff (*N* = 214) were randomised to MBSH, classical music, or waitlist conditions and completed self-report measures pre-, mid- and post-intervention. Post-intervention, MBSH was found to significantly reduce stress compared to both control conditions. Bootstrapping-based mediation analyses used standardised residualised change scores for all variables, with mediators computed as change from baseline to mid-intervention, and the outcome computed as change from baseline to post-intervention. Changes in mindfulness, self-compassion and worry were found to significantly mediate the effects of MBSH versus both control conditions on changes in stress. Findings suggest that cultivating mindfulness specifically confers benefits to stress and that these benefits may occur through improving theorised mechanisms.

Mindfulness is commonly defined as the quality of awareness that arises through intentionally observing the stream of moment-to-moment experience in an open, accepting and non-judgemental way (Kabat-Zinn 1994). Over the past few decades, cultivating mindfulness through mindfulness practice has received increasing attention. Much of this can be attributed to the development of mindfulness-based interventions (MBIs) in clinical contexts, of which the most widely employed are mindfulness-based stress reduction (MBSR; Kabat-Zinn 1982) and mindfulness-based cognitive therapy (MBCT; Segal et al. 2002, 2013). MBSR and MBCT are eight-session group-based interventions that teach participants mindfulness through a range of mindfulness practices and teacher-led discussion, with the intention of improving wellbeing and mental health. This intention is supported by findings from randomised controlled trials (RCTs). Compared to control conditions, MBIs are effective at improving a range of outcomes, such as quality of life, severity of anxiety and depressive symptoms, risk of depressive relapse, stress and chronic pain (e.g. Chiesa and Serretti 2009; Godfrin and van Heeringen 2010; Green and Bieling 2012; Grossman et al. 2007; Hofmann et al. 2010; Kuyken et al. 2016; Strauss et al. 2014).

In addition to the evidence base for the effectiveness of MBIs on clinically relevant outcomes, studies have started to investigate the mechanisms underlying their effects using mediation analysis, which examines the indirect effect of a treatment (X) on an outcome (Y) through a mediator (M), or intervening variable. Kazdin (2007) described a number of benefits of identifying how psychotherapies work, including the potential to better understand the outcomes of treatments, enhance aspects of interventions to optimise therapeutic benefits, facilitate the translation of research on treatments into practice and identify treatment moderators so that therapies can be matched to individuals. Recently, Gu et al. (2016a) systematically reviewed mediation studies examining the effects of MBSR and MBCT compared to control conditions on mental health and wellbeing outcomes and evaluated the strength of evidence for each identified mechanism. They found that most mediation studies selected mediators based on the theoretical underpinnings of these MBIs, with the most commonly tested mechanism being mindfulness, followed by repetitive negative thinking constructs (worry and rumination) and self-compassion. They identified moderate and consistent evidence for mindfulness and repetitive negative thinking as mechanisms, but insufficient evidence for self-compassion. However, many of the studies reviewed had at least one key methodological limitation. For example, most studies did not compare an MBI to a well-matched control condition. Comparing interventions to matched control conditions, which hold constant all factors except for the core, specific elements of the intervention, is important because this makes it possible to determine whether the specific elements of the intervention are responsible for beneficial outcomes (Mohr et al. 2009). Without adequately matched control conditions, it is possible that benefits are the result of non-specific elements of the intervention (e.g. expectation of benefit, facilitator contact, social support or interaction with group members) rather than specific elements (i.e. learning mindfulness).

Given the evidence base for the effectiveness of MBIs and emerging evidence illuminating their mechanisms, researchers have started to examine ways to increase the accessibility of MBIs to benefit more people. One way of extending the accessibility of interventions is to develop self-help versions (i.e. learning mindfulness through online courses, self-help books, smartphone applications, etc.). An additional benefit of mindfulness-based self-help (MBSH) interventions is that they remove many of the non-specific factors found in group-based MBIs, such as facilitator contact and group support and interaction, making them particularly suited to examining the effects of learning mindfulness specifically. Meta-analyses of RCTs of MBSH interventions and acceptance-based self-help interventions have shown beneficial effects on stress with a medium effect size, and depression, anxiety, wellbeing and mindfulness with small to medium effect sizes (Cavanagh et al. 2014; Spijkerman et al. 2016). However, these findings were based largely on combining data from studies which compared MBSH interventions to waitlist control conditions. If we truly want to examine the effects of learning mindfulness and the specific change mechanisms associated with these effects, we not only want an MBSH intervention that removes non-specific elements of face-to-face MBIs, we would also want a matched control intervention that controls for additional non-specific factors (Mohr et al. 2009), namely, expectation of benefit and engagement with a structured intervention of a similar format and with similar time demands. To our knowledge, only nine studies have compared MBSH interventions to matched control conditions (Carissoli et al. 2015; Dowd et al. 2015; Howells et al. 2016; Jimenez 2008; Ly et al. 2014; Mongrain et al. 2016; Niles et al. 2012; Stankovic 2015; Wahbeh et al. 2016). Of these studies, four examined online MBSH interventions, three audio CDs and two smartphone applications, and four compared MBSH to psychoeducation, two to relaxation exercises, one to behavioural activation, one to list making and one to expressive writing. None of these studies examined the mechanisms underlying the effects of learning mindfulness.

The primary aim of the present study was to examine whether a 2-week online MBSH intervention significantly reduces perceived stress compared to an inactive waitlist control condition and a matched non-mindfulness condition. The matched control condition consisted of a 2-week online classical music listening intervention, which shares the same structure, format and time demands as the MBSH intervention. We also aimed to test three theoretically and/or empirically supported mechanisms of MBIs (Gu et al. 2016a)—namely, mindfulness, self-compassion and worry—as mediators of the effects of MBSH versus the waitlist control and matched non-mindfulness conditions on stress. This study therefore investigates not only whether the specific process of learning mindfulness reduces stress, by comparing MBSH to a matched non-mindfulness condition and controlling for non-specific factors, but also how learning mindfulness specifically may reduce stress, by examining the three most commonly tested, and theorised, mechanisms of MBIs as mediators. This study tests three hypotheses. First, compared to the waitlist control condition, the MBSH intervention was predicted to significantly reduce stress over the 2-week time period. No hypothesis was made about the relative effect of the MBSH versus classical music intervention on stress, because there are no compelling theoretical or empirical reasons to hypothesise a difference between these conditions in either direction. Second, the effects of the MBSH intervention versus waitlist control on changes in stress were expected to be significantly mediated by changes in mindfulness, self-compassion and worry (Fig. 1a–c). Third, the effects of the MBSH intervention versus the classical music intervention on changes in stress were hypothesised to be significantly mediated by changes in mindfulness, self-compassion and worry (Fig. 1a–c).

**Fig. 1:**
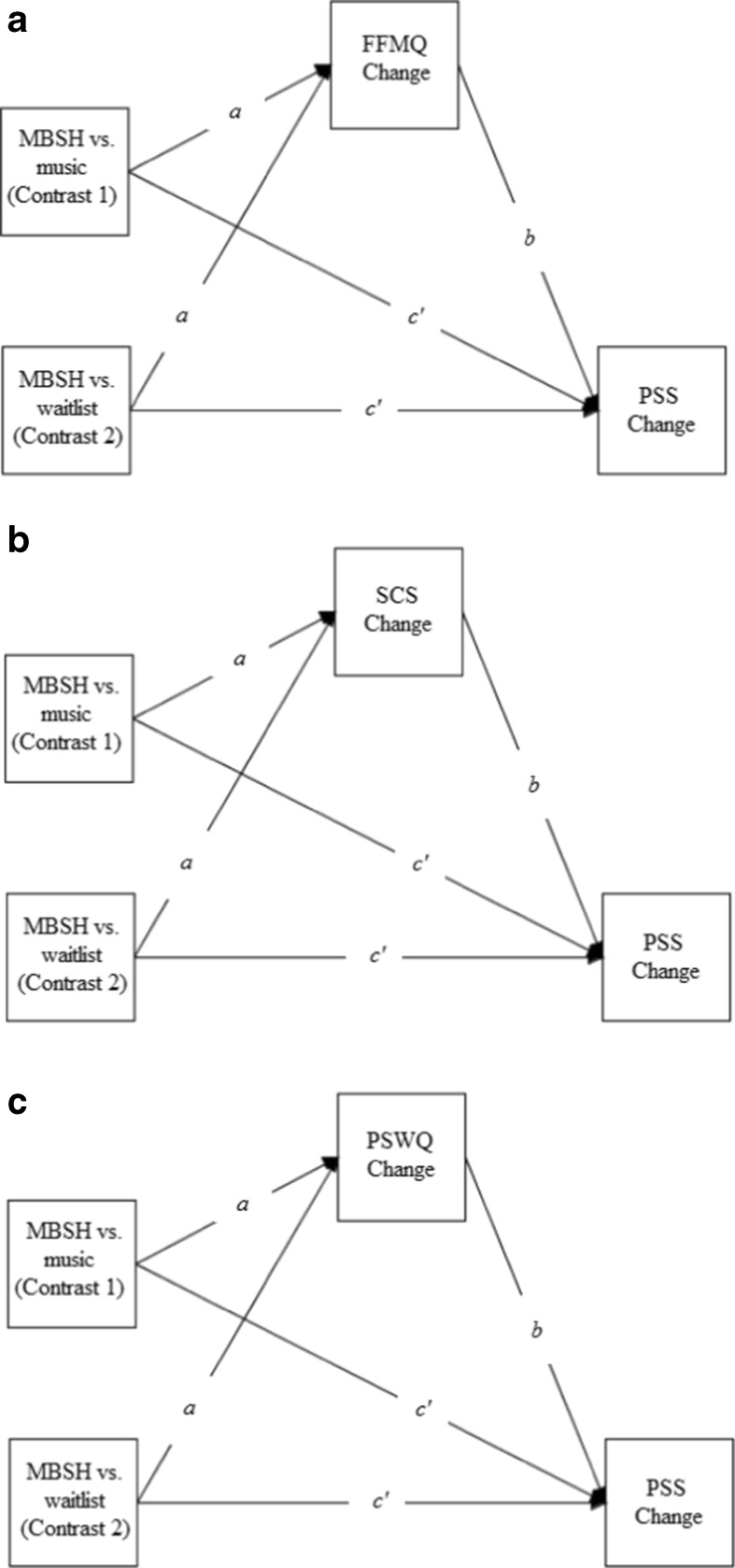
Path diagrams depicting three multicategorical independent variable mediation models. **a** The effects of mindfulness-based self-help (MBSH) versus music or MBSH versus waitlist control (X) on changes in perceived stress (PSS) (Y) mediated by changes in mindfulness (FFMQ) (M). **b** The effects of MBSH versus music or MBSH versus waitlist control on changes in perceived stress mediated by changes in self-compassion (SCS). **c** The effects of MBSH versus music or MBSH versus waitlist control on changes in perceived stress mediated by changes in worry (PSWQ). *a*, *b* and *c’* are unstandardised regression coefficients which represent predicting M from X (*a*), Y from M controlling for X (*b*) and Y from X controlling for M (*c’*). The product of the *a* and *b* paths, *ab*, represents the mediated or indirect effect

## Method

### Participants

This study had an experimental design, testing the effects of group (MBSH, classical music and waitlist control) and time (baseline, mid-intervention and post-intervention) on self-reported levels of mindfulness, worry, self-compassion and perceived stress. Participants were 214 students and staff (72.90% female) at a university in the South of England, with access to the university’s online learning portal. Their ages ranged from 18 to 49 years (*M* = 24.20, *SD* = 5.79). Participant baseline demographic characteristics are presented in Table 1.

**Table 1:** Baseline demographic characteristics of all participants across MBSH, classical music and waitlist control groups

Variable	Total (*N* = 214)		MBSH (*n* = 83)		Music (*n* = 68)		Waitlist (*n* = 63)	
	*M*	*SD*	*M*	*SD*	*M*	*SD*	*M*	*SD*
Age	24.20	5.79	24.94	6.89	24.38	5.76	23.02	3.81
Gender	*N*	%	*N*	%	*N*	%	*N*	%
Male	58	27.10	18	21.69	19	27.94	21	33.33
Female	156	72.90	65	78.31	49	72.06	42	66.67
Occupation								
Student	187	87.38	67	80.72	59	86.76	61	96.83
Staff	27	12.62	16	19.28	9	13.24	2	3.17
Length of mindfulness practice								
No experience	117	54.67	43	51.81	36	52.94	38	60.32
Less than a year	62	28.97	29	34.94	19	27.94	14	22.22
1–5 years	31	14.49	11	13.25	11	16.18	9	14.29
Over 5 years	4	1.87	0	0	2	2.94	2	3.17
Frequency of mindfulness practice								
Not at all	120	56.07	43	51.81	37	54.41	40	63.49
Once a month or less	60	28.04	26	31.33	20	29.41	14	22.22
About once a week	30	14.02	13	15.66	9	13.24	8	12.70
Most days	4	1.87	1	1.20	2	2.94	1	1.59
Length of CM listening								
No experience	96	44.86	33	39.76	32	47.06	31	49.21
Less than a year	26	12.15	9	10.84	12	17.65	5	7.94
1–5 years	27	12.62	13	15.66	7	10.29	7	11.11
Over 5 years	65	30.37	28	33.73	17	25.00	20	31.75
Frequency of CM listening								
Not at all	95	44.39	32	38.55	33	48.53	30	47.62
Once a month or less	76	35.51	37	44.58	19	27.94	20	31.75
About once a week	35	16.36	11	13.25	14	20.59	10	15.87
Most days	8	3.74	3	3.61	2	2.94	3	4.76

*CM* classical music, *MBSH* mindfulness-based self-help

### Procedure

Participants completed baseline questionnaires hosted on Bristol Online Surveys (www.onlinesurveys.ac.uk) and were randomised to the MBSH, classical music or waitlist control condition. Randomisation was conducted by researchers independent of the research team and blind to participant details using a computer-generated blocked allocation method, with six numbers per block. Participants allocated to MBSH and music conditions were given access to the intervention sites within 24 h of randomisation. Standardised e-mails were sent during the 2-week period (days 3, 7 and 10) to encourage participants to engage in the interventions. Participants were also e-mailed at days 7 (mid-intervention) and 14 (post-intervention) to complete online questionnaires. Reminder e-mails for participants to complete mid-intervention and post-intervention measures were sent once for mid-intervention measures and three times for post-intervention measures.

Participants randomised to the waitlist control condition were sent standardised e-mails within 24 h informing them of their allocation, and at days 7 and 14, asking them to complete mid- and post-intervention questionnaires. Due to limited study resources, it was not possible for researchers to be blind to condition allocation, but e-mail text was standardised to ensure that completion of questionnaires was not influenced by researcher bias. Participants were debriefed and given access to both online interventions upon completion of post-intervention questionnaires or after the 2-week waiting list period.

#### The Online Mindfulness and Music Interventions

The MBSH intervention, ‘Learning Mindfulness Online’, was taken from Cavanagh et al. (2013). The classical music intervention, ‘Listening to Classical Music Online’, mirrored the structure and format of the MBSH intervention as closely as possible, but differed in content. Both interventions were hosted on the university’s online learning portal.

#### Learning Mindfulness Online

This site consisted of eight pages. The welcome page provided information on what to expect from the intervention. *What is Mindfulness?* gave an overview of mindfulness and its benefits, history and practice using text and a brief video clip. *Daily Mindfulness Practice* contained a 10-min audio recording of a guided mindfulness practice and invited participants to listen to this for the duration of the intervention. Two versions of the recording were uploaded, one delivered by a female voice and one by a male voice, and participants could select which one they preferred to listen to. The audio clips were recorded by clinical psychologists trained to deliver MBIs. *Everyday Mindfulness Activities* described daily informal mindfulness practices. During the first week, participants were invited to bring mindfulness to one routine activity (e.g. showering), and during the second week, participants were invited to be mindful during a 5- to 30-min walk. The *Daily Practice and Everyday Mindfulness Activities FAQ* page provided advice on how to approach commonly encountered experiences during mindfulness practice (e.g. boredom, discomfort, sleepiness). Participants could use the *My Daily Journal* page to record their thoughts and feelings related to mindfulness practice. The *Study Information* page contained study information, and *Help and Assistance* gave the contact details of the research team, the university’s counselling service, and local and national mental health services.

#### Listening to Classical Music Online

This control site included eight matched pages: the welcome page, *Why Listen to Classical Music?*, *Daily Classical Music Listening*, *Everyday Musical Activities*, *Daily Listening and Everyday Musical Activities FAQ*, *My Daily Journal*, *Study Information* and *Help and Assistance*. The first five pages mirrored the MBSH site in terms of structure, but differed in the text, audio recordings and video used. References to mindfulness were replaced by information about classical music. Instead of the introductory video about mindfulness on the *What is Mindfulness?* page, the music site contained a brief introductory video about types of music and the potential benefits of listening to classical music. On the *Daily Classical Music Listening* page, two 10-min classical music pieces replaced the 10-min mindfulness recordings (Beethoven’s Piano Concerto No. 3 and No. 5). Consistent with previous research exploring the potential benefits of listening to classical music (e.g. Burns et al. 2002; Labbé et al. 2007), these pieces were selected based on their slow tempo markings. The final three pages were identical to the ones on the MBSH site.

### Measures

All measures were completed at baseline, mid-intervention and post-intervention with the exception of the plausibility question, which was completed at baseline only, the Engagement Questionnaire, which was completed at post-intervention only, and the Perceived Stress Scale (PSS), which was completed at baseline and post-intervention only. Cronbach’s alphas used baseline data from all participants (*N* = 214).

#### Five-Facet Mindfulness Questionnaire Short Form (FFMQ)

The 24-item FFMQ (Bohlmeijer et al. 2011) is a shortened version of the original 39-item measure (Baer et al. 2006). Both versions measure the general tendency to be mindful. The 24-item FFMQ is comprised of the same five facets as the original version: observing, describing, acting with awareness, non-judging of inner experience and non-reactivity to inner experience. Total scale and subscale scores of the 24-item FFMQ were found to be highly correlated with the 39-item version. Facets of the 24-item FFMQ were also found to be as similarly sensitive to change as the original measure. Items are rated on a five-point Likert scale ranging from 1 (never or very rarely true) to 5 (very often or always true). Consistent with the design of this study, participants were asked to complete this measure based on their experiences in the past week, and only the total FFMQ score was used. The total FFMQ scores at each time point did not include items from the observing facet, in line with recommendations for excluding this facet from comparisons of total FFMQ scores before and after mindfulness interventions (e.g. Gu et al. 2016b). This recommendation is based on findings demonstrating differences in FFMQ factor structure before and after mindfulness training in the same sample; before mindfulness training, a four-factor hierarchical model (without the observing facet) best fit the data, but after mindfulness training, a five-factor hierarchical model (with all five facets) best fit data (Gu et al. 2016b). Cronbach’s alpha in this study for the total FFMQ score (excluding observing items) was 0.87.

#### Self-Compassion Scale Short Form (SCS)

The 12-item SCS (Raes et al. 2011) is a shortened version of the original 26-item version (Neff 2003). Confirmatory factor analysis supported a six-factor hierarchical structure of the measure with the same six factors as the original version: self-kindness, self-judgement, common humanity, isolation, mindfulness and over-identification. However, internal consistency was variable for individual subscale scores and it is recommended that only the total SCS score is used. Items are rated on a five-point Likert scale from 1 (almost never) to 5 (almost always). Cronbach’s alpha in this study for the total SCS score was 0.83.

#### Penn State Worry Questionnaire (PSWQ)

The 16-item PSWQ (Meyer et al. 1990) is a widely used measure of trait worry. Each item is rated on a five-point Likert scale from 1 (not at all typical of me) to 5 (very typical of me). In the current study, participants completed this measure based on their experiences in the past week. Cronbach’s alpha in this study for the total PSWQ score was 0.91.

#### Perceived Stress Scale (PSS)

The ten-item PSS (Cohen et al. 1983) measures participants’ perceptions of situations in their life as stressful. Items ask participants to rate how often they have thought or felt in a certain way during the last month using a five-point Likert scale ranging from 0 (never) to 4 (very often). Participants completed this measure based on their experiences in the past week. Cronbach’s alpha in this study for the total PSS score was = 0.85.

#### Engagement Questionnaire

The engagement questionnaire, designed by the research team, consisted of the following four questions: (1) “Over the past two weeks, how much time in total have you spent using the online site, not including time spent listening to the audio recordings?”; (2) “Over the past two weeks, on how many days have you spent using the online site, not including time spent listening to the audio recordings?”; (3) “Over the past two weeks, how much time in total have you spent listening to the audio recordings?”; and (4) “Over the past two weeks, on how many days have you spent listening to the audio recordings?”. Participants answered questions 1 and 3 by entering the number of minutes and questions 2 and 4 by selecting a number from 0 to 14.

#### Plausibility Question

Participants were asked about their perceived plausibility of the MBSH and classical music interventions using the following question: “On a scale from 1 (not at all) to 9 (very much), how much do you feel that mindfulness meditation/classical music will help your wellbeing?”

### Data Analyses

#### Preliminary Analyses

Pearson’s chi-square test was conducted to examine between-group differences in the completion of questionnaires. Independent *t* tests were conducted to investigate baseline differences between completers and non-completers of all three sets of questionnaires. Pearson’s chi-square and one-way independent analysis of variance (ANOVA) were conducted to determine whether demographic variables differed between groups. Only participants who completed all three sets of questionnaires (baseline, mid-intervention and post-intervention) were included in the main and mediation analyses.

#### Hypothesis 1: Main Effects

A three (group: MBSH, music and waitlist) by two (time: baseline and post-intervention) mixed ANOVA was conducted on stress scores. A significant interaction effect was followed up with an analysis of covariance (ANCOVA) test and planned simple contrasts, to examine between-group differences in post-intervention stress scores controlling for baseline stress scores.

#### Hypotheses 2 and 3: Mediation Analyses

Mediation analyses used standardised residualised change scores for mediator and outcome variables. Standardised residuals were calculated using a linear regression model in which baseline scores predicted mid-intervention scores for mediators (mindfulness, self-compassion and worry) and baseline scores predicted post-intervention scores for the outcome variable (perceived stress). As the independent variable (IV), group, is multicategorical (with three levels: MBSH, classical music and waitlist) rather than dichotomous, three multicategorical IV mediation models were tested (Fig. 1), one for each mediator, as recommended by Hayes and Preacher (2014). In each model, two IV contrasts were examined using MBSH as the reference category: MBSH versus music and MBSH versus waitlist control. Testing multiple mediation models was unsuitable given the theoretical and empirical overlap between mindfulness and worry (e.g. Feldman et al. 2007; Verplanken and Fisher 2013) and mindfulness and self-compassion (e.g. Baer et al. 2012); this would test mindfulness as a mediator after controlling for worry and self-compassion as mediators (and vice versa), rather than the overall ability of mindfulness, self-compassion and worry as mediators (Preacher and Hayes 2008).

The three multicategorical IV models were tested using BC bootstrapping implemented in Mplus version 7.4 (Muthén and Muthén 1998–2015). Point estimates of each indirect effect (*ab*) were calculated by averaging the *ab* product from 5000 random samples of the original data. Indirect effects are significant if the upper and lower boundaries of the bootstrapped 95% BC confidence intervals (CIs) do not contain zero. Path coefficients were also calculated in Mplus for the effect of the IV on the mediator (path *a*), the effect of the mediator on the DV controlling for the IV (path *b*) and the direct effect of the IV on the DV controlling for the mediator (path *c’*). The path coefficients for the total effect of the IV on the DV *not* controlling for the mediator (path *c*) were calculated using linear regression in SPSS. Cohen’s *d* effect sizes were calculated for between-group (MBSH versus music, MBSH versus waitlist) effects on mediator and outcome variables (paths *a* and *c*).

## Results

**Fig. 2:**
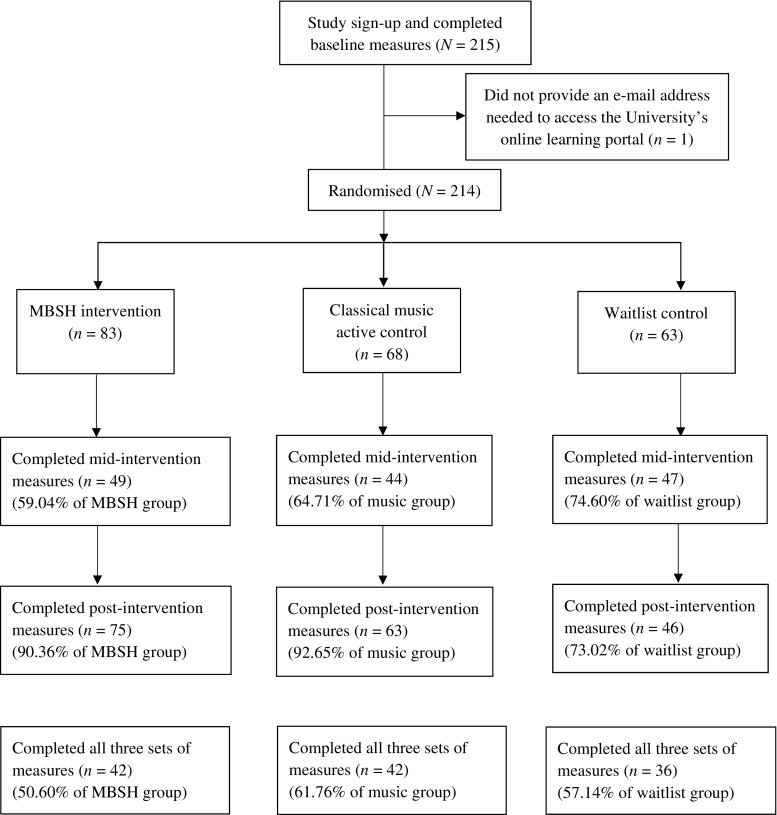
Participant flow through the study

Of the 214 participants randomised, 120 (56.07%) completed all three sets of measures and were included in the main and mediation analyses. Participant flow through the study is presented in Fig. 2. Table 2 shows the demographic characteristics of the completer sample; chi-square tests and a one-way ANOVA showed that the only demographic variable which significantly differed across groups at baseline was occupation.

**Table 2:** Baseline demographic characteristics of participants across MBSH, classical music and waitlist control groups who completed all three sets of questionnaires

Variable	Total (*n* = 120)		MBSH (*n* = 42)		Music (*n* = 42)		Waitlist (*n* = 36)		Statistics^a^
	*M*	*SD*	*M*	*SD*	*M*	*SD*	*M*	*SD*	
Age	24.66	6.40	25.64	8.10	24.33	6.23	23.89	3.85	*F*(2, 117) = 0.81, ns
Gender	*N*	%	*N*	%	*N*	%	*N*	%	*χ* ^2^(2) = 0.27, ns
Male	36	30	12	28.57	12	28.57	12	33.33	
Female	84	70	30	71.43	30	71.43	24	66.67	
Occupation									*χ* ^2^(2) = 6.48, *p* = .04
Student	109	90.83	35	83.33	38	90.48	36	100	
Staff	11	9.17	7	16.67	4	9.52	0	0	
Length of mindfulness practice									*χ* ^2^(6) = 6.90, ns
No experience	70	58.33	23	54.76	25	59.52	22	61.11	
Less than a year	28	23.33	13	30.95	9	21.43	6	16.67	
1–5 years	20	16.67	6	14.29	8	19.05	6	16.67	
Over 5 years	2	1.67	0	0	0	0	2	5.56	
Frequency of mindfulness practice									*χ* ^2^(6) = 2.66, ns
Not at all	71	59.17	23	54.76	25	59.52	23	63.89	
Once a month or less	27	22.50	9	21.43	10	23.81	8	22.22	
About once a week	20	16.67	9	21.43	7	16.67	4	11.11	
Most days	2	1.67	1	2.38	0	0	1	2.78	
Length of CM listening									*χ* ^2^(6) = 2.38, ns
No experience	52	43.33	16	38.10	20	47.62	16	44.44	
Less than a year	8	6.67	2	4.76	4	9.52	2	5.56	
1–5 years	16	13.33	7	16.67	4	9.52	5	13.89	
Over 5 years	44	36.67	17	40.48	14	33.33	13	36.11	
Frequency of CM listening									*χ* ^2^(6) = 4.53, ns
Not at all	50	41.67	15	35.71	19	45.24	16	44.44	
Once a month or less	46	38.33	21	50.00	14	33.33	11	30.56	
About once a week	20	16.67	5	11.90	8	19.05	7	19.44	
Most days	4	3.33	1	2.38	1	2.38	2	5.56	

*CM* classical music, *MBSH* mindfulness-based self-help

^a^Statistical test for between-group differences in demographic characteristics of participants at baseline. The only demographic variable which significantly differed across groups at baseline was occupation

Completion rates were not found to significantly differ across groups (*χ* ^2^(2) = 1.93, *p* = .381). Chi-square tests with Bonferroni-corrected alpha levels of 0.0083 (0.05/6) showed no significant differences between completers and non-completers in gender, occupation, classical music experience (length of experience and frequency of listening to music) and mindfulness experience (length of experience and frequency of mindfulness practice). Independent *t* tests with Bonferroni-corrected alpha levels of 0.01 (0.05/5) demonstrated no significant differences between completers and non-completers in terms of age and baseline self-compassion, worry and stress scores. However, completers were found to have significantly higher baseline mindfulness scores (*M* = 77.34, *SD* = 10.66) compared to non-completers (*M* = 73.57, *SD* = 8.91) (*p* = .005). Mean baseline, mid-intervention and post-intervention scores for all measures across groups and time points are shown in Table 3.

**Table 3:** Mean total mindfulness, self-compassion, worry and perceived stress scores across all conditions and time points in the completer sample (*n* = 120)

	MBSH (*n* = 42)			Music (*n* = 42)			Waitlist (*n* = 36)		
	Pre	Mid	Post	Pre	Mid	Post	Pre	Mid	Post
FFMQ^a^	62.48 (10.05)	67.10 (10.82)	67.45 (11.02)	63.26 (10.00)	64.07 (10.27)	65.90 (9.96)	64.11 (11.18)	64.89 (11.80)	64.75 (13.36)
SCS	33.86 (9.24)	37.38 (8.80)	37.83 (8.12)	35.10 (7.75)	36.00 (7.10)	37.64 (7.40)	36.94 (9.31)	37.11 (10.29)	37.25 (10.63)
PSWQ	53.79 (12.29)	47.26 (11.80)	47.00 (10.82)	51.19 (11.98)	50.55 (11.32)	49.48 (11.17)	51.78 (15.43)	51.33 (15.21)	49.47 (17.02)
PSS	28.88 (7.41)	–	25.48 (6.63)	28.83 (6.84)	–	28.31 (7.06)	27.97 (8.27)	–	28.33 (7.90)

Standard deviations are shown in parentheses

*FFMQ* Five-Facet Mindfulness Questionnaire, *MBSH* mindfulness-based self-help, *PSS* Perceived Stress Scale, *PSWQ* Penn State Worry Questionnaire, *SCS* Self-Compassion Scale

^a^Mean total FFMQ scores do not include items from the observing facet

### Hypothesis 1: Main Effects

Mixed ANOVA showed a significant group by time interaction for perceived stress, *F*(2, 117) = 3.35, *p* = .038. Follow-up ANCOVA showed a significant effect of group on post-intervention stress scores controlling for baseline stress scores, *F*(2, 116) = 3.77, *p* = .026; post-intervention stress scores were significantly lower in the MBSH group compared to the waitlist control (contrast estimate = 3.35, *p* = .014) and in the MBSH compared to the music group (contrast estimate = 2.86, *p* = .029). These findings supported hypothesis 1.

### Hypotheses 2 and 3: Mediation Analyses

**Fig. 3:**
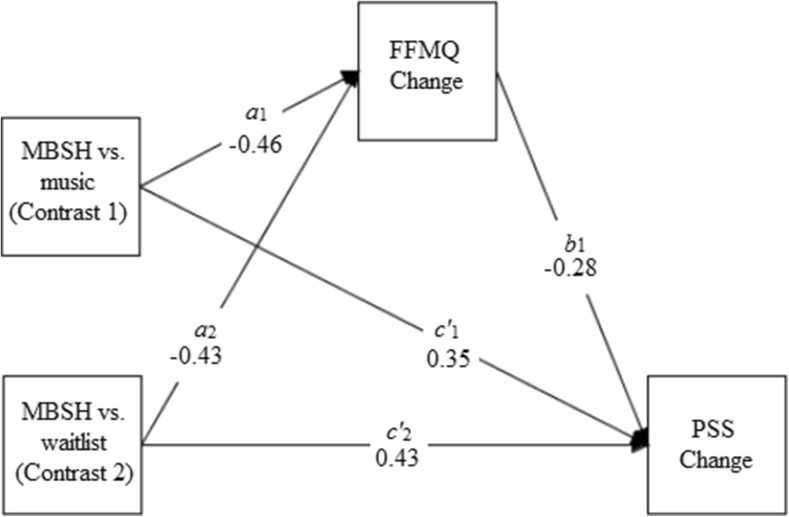
Path diagram depicting model 1, testing whether changes in mindfulness (FFMQ change) mediate the effects of mindfulness-based self-help (MBSH) versus music (contrast 1) or MBSH versus waitlist control (contrast 2) on improvements in perceived stress (PSS change). Unstandardised path coefficients are displayed. Change refers to standardised residualised change scores

**Fig. 4:**
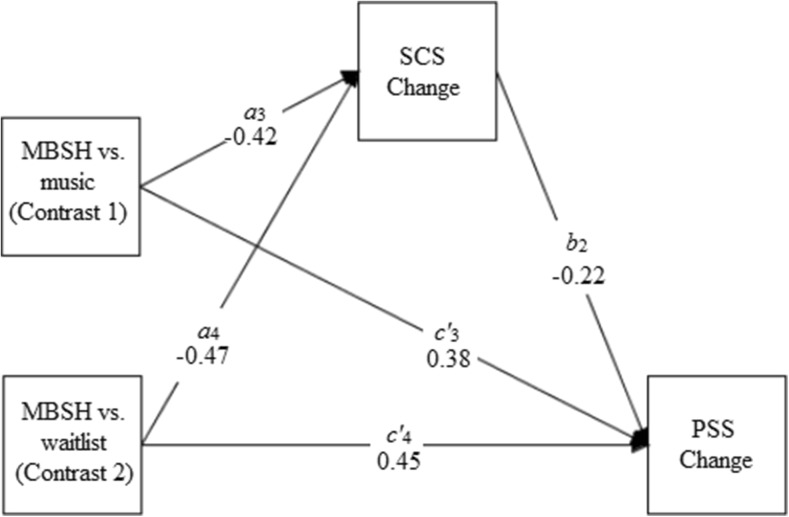
Path diagram depicting model 2, testing whether changes in self-compassion (SCS change) mediate the effects of mindfulness-based self-help (MBSH) versus music (contrast 1) or MBSH versus waitlist control (contrast 2) on improvements in perceived stress (PSS change). Unstandardised path coefficients are displayed. Change refers to standardised residualised change scores

**Fig. 5:**
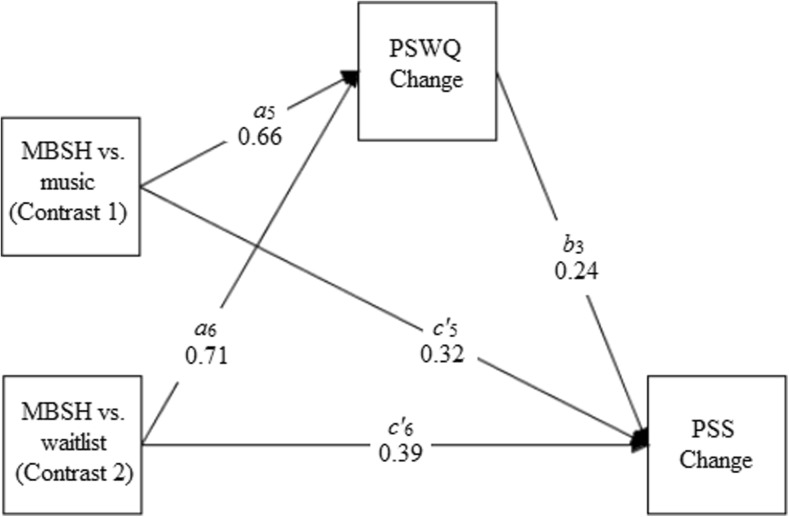
Path diagram depicting model 3, testing whether changes in worry (PSWQ change) mediate the effects of mindfulness-based self-help (MBSH) versus music (contrast 1) or MBSH versus waitlist control (contrast 2) on improvements in perceived stress (PSS change). Unstandardised path coefficients are displayed. Change refers to standardised residualised change scores

Table 4 presents the results of the mediation analyses for the three multicategorical IV mediation models that tested whether standardised residualised change scores in mindfulness, self-compassion and worry mediated the relationship between group (MBSH vs. waitlist, MBSH vs. music) and standardised residualised change scores in perceived stress. Figures 3, 4 and 5 present path diagrams of these models.

**Table 4:** Unstandardised regression coefficients, their standard errors (SEs) and significance values, and bootstrapped unstandardised point estimates and their SEs and 95% bias-corrected confidence intervals, for the three multicategorical independent variable mediation models

Model	*B*	*SE*	*t*	*p*	Point estimate (*SE*) [95% BC CIs]^a^
Model 1. With *mindfulness* as the mediator.					
Contrast 1. Group: MBSH vs. music					0.13 (0.08) [0.01, 0.15]
*a*_1_ path: group → FFMQ change	− 0.46	0.22	− 2.06	.040	
*b*_1_ path: FFMQ change → PSS change	− 0.28	0.09	− 3.15	.002	
*c*_1_ path: group → PSS change	0.47	0.23	2.10	.039	
*c’*_1_ path: group → PSS change (direct effect)	0.35	0.22	1.58	.114	
Contrast 2. Group: MBSH vs. waitlist control					0.12 (0.08) [0.002, 0.15]
*a*_2_ path: group → FFMQ change	− 0.43	0.23	− 1.88	.060	
*b*_1_ path: FFMQ change → PSS change	− 0.28	0.09	− 3.15	.002	
*c*_2_ path: group → PSS change	0.28	0.10	2.72	.008	
*c’*_2_ path: group → PSS change (direct effect)	0.43	0.19	2.28	.023	
Model 2. With *self-compassion* as the mediator.					
Contrast 1. Group: MBSH vs. music					0.09 (0.06) [0.01, 0.25]
*a*_3_ path: group → SCS change	− 0.42	0.21	− 1.99	.047	
*b*_2_ path: SCS change → PSS change	− 0.22	0.09	− 2.54	.011	
*c*_1_ path: group → PSS change	0.47	0.23	2.10	.039	
*c’*_3_ path: group → PSS change (direct effect)	0.38	0.23	1.67	.094	
Contrast 2. Group: MBSH vs. waitlist control					0.10 (0.07) [0.01, 0.28]
*a*_4_ path: group → SCS change	− 0.47	0.24	− 2.00	.045	
*b*_2_ path: SCS change → PSS change	− 0.22	0.09	− 2.54	.011	
*c*_2_ path: group → PSS change	0.28	0.10	2.72	.008	
*c’*_4_ path: group → PSS change (direct effect)	0.45	0.20	2.30	.021	
Model 3. With *worry* as the mediator.					
Contrast 1. Group: MBSH vs. music					0.16 (0.07) [0.05, 0.33]
*a*_5_ path: group → PSWQ change	0.66	0.21	3.23	.001	
*b*_3_ path: PSWQ change → PSS change	0.24	0.09	2.58	.010	
*c*_1_ path: group → PSS change	0.47	0.23	2.10	.039	
*c’*_5_ path: group → PSS change (direct effect)	0.32	0.23	1.38	.166	
Contrast 2. Group: MBSH vs. waitlist control					0.17 (0.08) [0.05, 0.37]
*a*_6_ path: group → PSWQ change	0.71	0.21	3.43	.001	
*b*_3_ path: PSWQ change → PSS change	0.24	0.09	2.58	.010	
*c*_2_ path: group → PSS change	0.28	0.10	2.72	.008	
*c’*_6_ path: group → PSS change (direct effect)	0.39	0.19	2.00	.045	

Standardised residualised change scores were used for all mediator and outcome variables

*BC CIs* bias-corrected confidence intervals, *MBSH* mindfulness-based self-help, *PSS* Perceived Stress Scale, *PSWQ* Penn State Worry Questionnaire, *SCS* Self-Compassion Scale

^a^Bootstrapped 95% BC CIs for the *ab* (indirect) effect; a significant indirect effect is indicated where these do not cross zero (*p* < .05)

#### Hypothesis 2

Participation in MBSH versus waitlist control on change in perceived stress was hypothesised to be significantly mediated by improvements in mindfulness, self-compassion and worry. Change in mindfulness was found to significantly mediate the effects of participating in MBSH versus waitlist on changes in stress (model 1, contrast 2), as indicated by the bootstrapped 95% BC CI which did not cross zero (Table 4). All path coefficients apart from the one corresponding to path *a*_2_ were significant. This shows that there was a significant and moderate effect of group (MBSH versus waitlist) on change in stress (path *c*_2_) in favour of the MBSH group (*d* = 0.62) and that change in mindfulness was a significant predictor of change in stress (path *b*_1_). Although the effect of group on change in mindfulness just failed to reach statistical significance (path *a*_2_; *p* = .060), there was a moderate effect size (*d* = 0.43) in favour of the MBSH group.

Change in self-compassion was found to be a significant mediator of the effects of MBSH versus waitlist on change in stress (model 2, contrast 2), as indicated by the bootstrapped 95% BC CI which did not include zero. All paths in this model were significant. In addition to the significant, moderate effect of group (MBSH versus waitlist) on changes in stress (path *c*_2_; *d* = 0.62), there was a significant, moderate effect of group on change in self-compassion (path *a*_4_) in favour of the MBSH group (*d* = 0.45) and change in self-compassion was a found to be a significant predictor of changes in stress (path *b*_2_).

The effects of MBSH versus waitlist on change in stress was significantly mediated by changes in worry (model 3, contrast 2), as indicated by the bootstrapped 95% BC CI which did not include zero. All paths in this model were significant. In addition to the significant, moderate effect of group (MBSH versus waitlist) on change in stress (path *c*_2_; *d* = 0.62), there was a significant, large effect of group on change in worry (path *a*_6_), in favour of the MBSH group (*d* = 0.77), and change in worry was found to be a significant predictor of change in stress (path *b*_3_). Taken together, these results supported hypothesis 2.

#### Hypothesis 3

Participation in the MBSH versus classical music condition on change in perceived stress was predicted to be significantly mediated by improvements in mindfulness, self-compassion and worry. Change in mindfulness was found to significantly mediate the effect of participating in MBSH versus music on changes in perceived stress (model 1, contrast 1), as indicated by bootstrapped 95% BC CIs which did not contain zero. All regression coefficients apart from the one corresponding to path *c’*_1_ were significant. In addition to change in mindfulness being a significant predictor of change in stress (path *b*_1_), there was a significant, moderate effect of group (MBSH versus classical music) on change in stress (path *c*_1_), in favour of the MBSH group (*d* = 0.46), and significant, moderate effect of group on change in mindfulness (path *a*_1_) in favour of the MBSH group (*d* = 0.46).

Change in self-compassion was found to significantly mediate the effects of MBSH versus music on change in stress (model 2, contrast 1), as indicated by bootstrapped CIs which did not cross zero. All paths in this model apart from the one corresponding to path *c’*_3_ were significant. In addition to change in self-compassion being a significant predictor of change in stress (path *b*_2_) and a significant, moderate effect of group (MBSH versus classical music) on change in stress (path *c*_1_; *d* = 0.46), there was a significant, moderate effect of group on change in self-compassion (path *a*_3_) in favour of the MBSH group (*d* = 0.43).

Change in worry was found to significantly mediate the effects of MBSH versus music on change in stress (model 2, contrast 1), as indicated by bootstrapped CIs which did not include zero. All paths in this model apart from the one corresponding to path *c’*_5_ were significant. In addition to change in worry being a significant predictor of change in stress (path *b*_3_) and a significant, moderate effect of group (MBSH versus classical music) on change in stress (path *c*_1_; *d* = 0.46), there was a significant, moderate-large effect of group on change in worry (path *a*_5_) in favour of the MBSH group (*d* = 0.70). Taken together, these findings supported hypothesis 3.

### Intervention Engagement

There were no significant differences between the MBSH and music conditions on any of the four engagement indices: time spent browsing the site (*t*(82) = 0.35, *p* = .724), number of days spent browsing the site (*t*(75.18) = 1.46, *p* = .148), time spent listening to audio recordings (*t*(82) = 1.07, *p* = .286) and number of days spent listening to audio recordings (*t*(82) = 0.34, *p* = .732).

Participants in the MBSH condition reported spending an average of 72.98 min (*SD* = 63.93) and 5.12 days (*SD* = 3.66) over the 2 weeks browsing the online site, not including time spent listening to audio recordings. They reported spending on average 99.43 min (*SD* = 83.64) and 7.52 days (*SD =* 4.52) over the 2 weeks listening to audio recordings on the site.

Participants in the classical music condition reported spending an average of 79.05 min (*SD* = 90.86) and 4.10 days (*SD* = 2.69) over the 2 weeks browsing the online site, not including time spent listening to audio recordings. They reported spending on average 121.33 min (*SD* = 102.47) and 7.21 days (*SD =* 3.68) over the 2 weeks listening to audio recordings on the site.

### Intervention Plausibility

For the completer sample, there was no significant difference in perceived plausibility of received intervention between participants randomised to the MBSH intervention (*M =* 5.74, *SD* = 2.04) and participants randomised to the music intervention (*M =* 5.02, *SD* = 1.99), *t*(82) = 1.62, *p* = .108.

## Discussion

This experimental study examined the effects of a 2-week online MBSH intervention on perceived stress compared to matched classical music and inactive waitlist control groups. This study also tested whether improvements in three theoretically and/or empirically supported mechanisms of MBIs (mindfulness, self-compassion and worry) mediate the effects of MBSH compared to both control conditions on changes in stress.

Consistent with hypothesis 1, MBSH was found to significantly reduce stress at post-intervention compared to both the matched classical music condition and the waitlist condition. In addition, MBSH had moderate-to-large effects on improvements in not only stress, but also mindfulness, self-compassion, and worry over the course of the intervention (indicated by coefficients for paths *a* in the mediation analyses), in comparison to both control conditions. Findings support and extend the modest body of evidence for the effectiveness of MBSH interventions (Cavanagh et al. 2014). Given that listening to classical music was rated by participants as equally plausible and engaging as MBSH, the finding that MBSH had significant effects on stress compared to the music intervention suggests that MBSH may be a particularly effective way of managing stress in a non-clinical population.

Both mediational hypotheses were supported; improvements in mindfulness, self-compassion and worry significantly mediated the effects of MBSH on changes in stress in comparison to a waitlist control group and a matched, equally plausible and engaging classical music intervention. This suggests that the mediating effects of mindfulness, self-compassion and worry on stress outcomes are specific to learning mindfulness and not general features of any plausible self-help intervention. The inclusion of a well-matched control group allows stronger inferences to be made regarding specificity of effects to MBSH.

These mediation findings suggest that there is an overlap between the mechanisms underlying MBIs and MBSH interventions; current findings are consistent with findings identifying mindfulness and worry as two of the most empirically supported mediators of the effects of MBIs on mental health outcomes (Gu et al. 2016a). Findings also support the theoretical literature on MBIs; evidence for mindfulness as a mediator supports the theoretical premise of MBIs such as MBCT and MBSR, that cultivating mindfulness improves mental health outcomes (Kabat-Zinn 1982; Segal et al. 2002, 2013). Findings are also consistent with the notion that self-compassion is embedded in mindfulness practice and crucial to the change process (Feldman and Kuyken 2011). Evidence for worry as a mediator supports MBCT theory that improved mental health outcomes, in particular reduced depressive relapse, are a result of decreasing maladaptive styles of thinking (Segal et al. 2002, 2013).

### Strengths and Limitations

This study addressed an important omission in the MBI literature investigating effectiveness and mechanisms. The use of MBSH allowed us to examine the specific effects associated with learning mindfulness, by removing many of the non-specific factors found in group-based MBIs (e.g. facilitator support, group process) and MBIs which incorporate cognitive behavioural therapy elements (e.g. MBCT) and stress reduction strategies (e.g. MBSR). By comparing MBSH with a well-matched non-mindfulness intervention, reported to be comparably plausible and engaging, this study also controlled for additional non-specific factors (e.g. expectation of benefit). This allows for stronger conclusions regarding the specificity of effects of learning mindfulness (i.e. mediating effects are not simply features of any plausible self-help intervention).

However, study attrition was high, with only 56.07% of participants completing measures at all three time points. Although not unusual in studies of online MBSH (e.g. Cavanagh et al. 2013), relatively low rates of study completion (i.e. completing measures at all three time points) can be largely attributed to participants who did not complete measures mid-intervention. Low rates of measure completion at mid-intervention may be because measures needed to be completed within a short time frame and as a result, only one reminder email was sent. Future studies incorporating assessment points during a brief intervention could inform participants of upcoming assessments (e.g. 24 h prior to sending questionnaires) in addition to sending multiple reminder e-mails.

Although the current study contributes to our understanding of how learning mindfulness might reduce stress, a more robust test of the underlying mechanisms would involve determining the temporal order of mediator and outcome variables, by assessing these variables during the intervention (e.g. at mid-intervention) and testing whether change in mediators predates change in outcomes (Kazdin 2007). In the current study, mindfulness, self-compassion and worry were found to be significant mediators of MBSH’s specific effects on stress, but we cannot infer causal direction from our data, because we do not know if changes in the proposed mediators improved prior to or following changes in stress. Future research should endeavour to include assessment of mediator and outcome variables during intervention in order to more closely examine temporal ordering and make more definitive conclusions regarding the causal mechanisms of MBIs.

Future studies should also follow up participants to determine whether improvements in outcomes gained from participating in MBSH are maintained beyond the intervention period. Further research may additionally benefit from using objective measures of engagement (e.g. number of times web pages and audio recordings were accessed) and including non-self-report measures of mediator and outcome variables.

Current findings show that online MBSH interventions may be promising alternatives to MBIs in non-clinical settings and in situations where resources are limited. However, a cautious approach to applying these findings to clinical practice should be taken. There is currently very limited evidence that MBSH can be offered safely and effectively in clinical settings (although see Dimidjian et al. 2014 for a recent promising example). As such, we do not recommend at present that MBSH, offered without support from a qualified mindfulness teacher, should be routinely offered in clinical settings. Further research is needed to test the safety, acceptability and effectiveness of MBSH in clinical populations and examine whether similar mechanisms are involved. MBSH in clinical settings may also require the addition of clinician guidance and support in order to maximise engagement and improve outcomes, as has been found for self-help CBT in these settings (cf. Gilbody et al. 2015).

Previous research examining the effects of MBIs and mechanisms of change has been hampered by failure to control for non-specific effects such as group process, facilitator support and expectation of benefit. This means that it remained possible that the benefits of MBIs could have been attributable, perhaps entirely, to these non-specific factors. Findings from the current study however suggest that mindfulness-specific factors do contribute, at least in part, to beneficial outcomes and change processes. Compared to a waitlist control group and a well-matched non-mindfulness condition, designed to control for non-specific effects, MBSH led to significant improvements in stress. Moreover, changes in mindfulness, self-compassion and worry significantly mediated the effects of MBSH versus both control conditions on stress reduction. Future research on mechanisms should include multiple assessments of mediator and outcome variables during intervention to draw robust conclusions regarding direction of causality. These findings demonstrate that learning mindfulness per se may confer specific benefits to mental health, through improving mindfulness and self-compassion and reducing worry.
